# Meeting the needs of underserved populations: setting the agenda for more inclusive citizen science of medicine

**DOI:** 10.1136/medethics-2018-105253

**Published:** 2019-07-12

**Authors:** Amelia Fiske, Barbara Prainsack, Alena Buyx

**Affiliations:** 1 Institute for History and Ethics in Medicine, Technical University of Munich, Munich, Germany; 2 Anthropology Department, University of North Carolina at Chapel Hill, Chapel Hill, North Carolina, USA; 3 Department of Political Science, University of Vienna, Vienna, Austria; 4 Department of Global Health & Social Medicine, King’s College London, London, UK

**Keywords:** citizen science, participant-led research, participation, biomedicine, medical research, medical ethics

## Abstract

In its expansion to genomic, epidemiological and biomedical research, citizen science has been promoted as contributing to the democratisation of medical research and healthcare. At the same time, it has been criticised for reinforcing patterns of exclusion in health and biomedicine, and sometimes even creating new ones. Although citizen science has the potential to make biomedical research more inclusive, the benefits of current citizen science initiatives are not equally accessible for all people—in particular those who are resource-poor, located outside of traditional networks of healthcare services, or members of minorities and marginalised groups. In view of growing public investments in participatory research endeavours, we argue that it should be considered more explicitly if, and how, citizen science could help make research more inclusive, contribute to the public good, and possibly even lead to better and more equitable healthcare. Reflecting on emerging ethical concerns for scientific conduct and best medical practice, we propose a set of relevant considerations for researchers, practitioners, bioethicists, funders and participants who seek to advance ethical practices of citizen-led health initiatives, and address profound differences in position, privilege and power in research.

While the phenomenon of non-professional experts contributing to scientific knowledge creation is by no means a novelty of recent decades, the rapid advancement of digital technologies has fuelled the expansion of ‘citizen science’ into wider areas of medicine, genomics, epidemiology and public health.^1^ The expansion of citizen science into areas of medical research has been framed as an important contribution to the democratisation of research^2^ adding new perspectives and value to clinical research,^3^ and to healthcare more broadly.^4^ Citizen science has borne hopes of developing treatments for rare diseases, advancing public health directives and extending recruitment in biomedical research,^5 6^ making it an increasingly central component of medical and health research and practice. The use of citizen science in medicine is also seen to bear the potential to make research more democratic and inclusive, for example by transforming hierarchies of expert and lay participation in knowledge production.

At the same time, the expansion of citizen-led practices in health and medicine raises ethical and political concerns. For example, it may confound and replicate existing power structures, exclusions and disparities in access to health resources, knowledge and technologies. Some have called for a new social contract to govern the production of health knowledge in novel configurations,^7^ while others have argued for more symmetrical engagements between biomedical ethicists, scientists and publics.^8^ In view of growing public investments in citizen-led endeavours, we argue that in addition to such approaches, we need to consider more explicitly whether, and how, citizen science could help make research more inclusive, foreground the aim of contributing to the public good, and possibly even lead to better and more equitable healthcare. Building on a background of established principles for ethical scientific conduct, best research practices and medical ethics,^9–11^ our prior work on ethics, participation, motivation and responsibility in citizen science,^1 6 12–16^ and responding to scholarship on emerging issues of diversity and inclusion in biomedical research and practice,^2 7 17–20^ we propose a set of considerations for researchers, practitioners, biomedical ethicists, funders and participants alike. The aim of these considerations is to advance ethical practices of citizen-led health initiatives, and address profound differences in position, privilege and power in research.

## New promises and new concerns

Citizen science—understood broadly as the participation of non-professionals at any phase of scientific research—has been celebrated for generating creative synergies of lay and expert collaboration to address health concerns.^21 22^ It is now easier than ever before for many lay individuals to participate in biomedical research. Popular news accounts describe patient-led research and how ‘Citizen science effort is empowering communities to advance health equity'.^23^ Many initiatives embrace the rhetoric of participation and democratisation, framing their work as ‘Personal health data for the public good’ (Health Data Exploration Project), or encouraging individuals to ‘Become a research partner. You can help make a difference’, (mPower), speaking to debates over whether an ethical duty to participate in health data research exists.^24^ The collection and sharing of personal data is promoted in many citizen-led projects as a means to facilitate advances in health for a broader community. Common types of citizen science range from gamified analytical tasks (eg, Eyewire, Foldit) to web-based self-recruiting in data-intensive projects (eg, Open Humans, PatientsLikeMe, Smart Patients). Other projects are dedicated to advancing research on rare conditions (eg, MyDaughtersDNA, DIYGenomics), compiling biorepositories (eg, American Gut Project, Healthbank) or recruiting citizens as data sensors (eg, Influenza near you, Mosquito Alert). In many cases, knowledge creation in the strict sense of the word, and research and project funding, are crowdsourced (eg, YouCaring, SciFund).^13^

Yet, not everyone has been so enthusiastic. Critiques include that ‘participation’ is limited to specific tasks, rarely involves the formulation of hypotheses or theory, or only refers to the use of lay individuals’ biological material.^25^ Initiatives tend to be used by a relatively select group of people, in particular those who are highly educated and already working in science or outreach spheres.^14 26^ Many projects suffer from demographic biases similar to ‘traditional’ medical research^27 28^ or speak only ‘about’ rather than ‘with’ vulnerable groups.^29^ When those who previously were referred to as ‘research subjects’ are now understood to be ‘participants’ and ‘proactive managers’ of their own health,^5^ the resulting research endeavours could be subject to less rigorous oversight intended to protect the rights of patient-subjects.^30^ In sum, there is growing concern that participatory initiatives in health and medicine do not always fulfil the promises of citizen science regarding more democratic and inclusive research practices.

While digital technologies and engagement possibilities through smartphones, wearables, handheld devices and other tools are rapidly changing research practices, ethical frameworks to navigate the challenges of participatory practices have not been sufficiently developed, particularly in medicine. Some citizen projects are initiated and run by lay individuals, while others are started by research groups, non-profits and companies, and then publics are invited to contribute. In this article, we use the term citizen science as an umbrella term that captures a broad class of practices that encompass many different types of research, each with its own objectives, stakeholders and ethical challenges. We do not attempt to classify the citizen science projects discussed here on the basis of their inputs, ‘bottom-up’ and ‘top-down’ approaches, or stated goals because many of these are often blurred in practice (for further work on this and a typology of citizen science initiatives please see the study by Prainsack *et al* 15). Indeed, one challenge that has accompanied citizen science as a growing field of participatory practices in health research is its high level of heterogeneity; while underscoring the range of practices at hand, we find that it is still productive to speak of a ‘field’—although a broad and dynamic one—of citizen science of medicine in order to address key ethical and political concerns with this growing area of research.^1^ We believe that ethical aspects should always be explored and addressed within the specific context of the project, its stated goals, participants and relevant history.

To aid such explorations, we propose a set of concerns that specifically address how citizen science of medicine, epidemiology, genomics and public health meets, or does not meet, the needs of underserved populations. Given the diversity of initiatives subsumed under the umbrella term of citizen science, we develop recommendations to guide further practice and explore ethical concerns raised in citizen science of health (table 1). We do this by building on established frameworks of research and medical ethics,^9–11^ and by reflecting on debates in research and medical ethics, personalised medicine, and critical public health. We focus on consistent concerns specific to citizen-led initiatives, such as diversity, inclusion and representation, that have not yet been fully addressed by traditional research ethics frameworks nor by citizen science efforts, and on the implications of current practices ranging from initiatives in public health, to crowdsourcing, to patient-led research in order to address a broad and ever-growing field of citizen science. In line with calls to address social problems such as inequity, racism and bias in biomedical ethics,^31^ we argue that researchers, practitioners, bioethicists, funders and participants in citizen science initiatives should work to promote more inclusive, open and equitable forms of engagement with health research.

**Table 1:** Questions to ask for those designing, running and funding citizen science initiatives

	*Concern*	*Questions to ask:*
Conditions, barriers, and burdens of participation	1. Who can participate?	How does our project actively address disparities in position, access, experience or resources? What explicit codes of conduct do participants agree on, and how are they determined? Do our website descriptions include visual aids and multimedia pieces?
	2. What are the barriers?	What fees or conditions (tools, skills, software, time, training) are necessary for participation? How do these conditions shape the resulting data set? Do website descriptions include visual aids and multimedia pieces that explain in plain language the aims of the project, and how to participate? What forms of literacy (scientific, medical, digital, English language) are required?
	3. What are the burdens of engagement?	What responsibilities or burdens are borne by participants? Were any of the services offered by the initiative previously provided by other groups, organisations or states? If so, are they still available?
Benefits and distribution of results	4. Who should benefit, and how?	Who is the intended public, how can they benefit? Who is empowered or disempowered in the process, and how? What specific, local benefits (as opposed to broad, global claims) are provided?
	5. How are results distributed	How can we make our products of better service to (wider ranges of) the public? Are results and tools open access? Are there other barriers to access? Are the products or end results, whenever possible, made from affordable, easy to use materials? Are the necessary instructions also shared to facilitate use?
Representation and recognition	6. What data are available to participants and publics?	What are the enabling or limiting conditions of data access and use? Are data contextualised, and what further provisions are necessary for data to be intelligible or useful? Are participants compensated for their labour or other contributions? Is data access feasible for marginalised groups or for those living in resource-poor areas? What benefits (as well as costs and risks) does data access entail for them?
	7. Who do the data represent?	Are members of marginalised groups involved in developing research aims and recruitment strategies? If working with members of marginalised or indigenous groups, are the participants co-collaborators on the project? Do they retain determination over final use of results, data or products? What markers of difference are used? How are they determined? How often are questions of representation revisited during our project?
Trust, risk and global inequalities	8. Recognise historic injustices, avoid repeating	Do relevant histories of gender-related, racial and colonial violence inform our project, and if so, which ones, and how? Does our initiative publicly recognise how it may benefit from, replicate or operate within institutions of structural violence and exclusion, and take steps to avoid repeating this in the future?
	9. Think about trust and be trustworthy	Who is likely to ‘opt-in’ to the project, and who is not? What role does trust play in this process? Are there pertinent histories of marginalisation that might influence the groups choosing to participate and those that do not? What can we do to increase the trustworthiness of our initiative in meaningful ways?
	10. Consider global justice	Could the collection of samples, reporting of real time events, or posting of personal data online pose personal harms to individuals, particularly those in poorer regions of the world? Are there any possible harms that could materialise along the global socioeconomic gradient, of particular relevance for marginalised, resource-poor populations?

## Conditions, barriers and burdens of participation

Even in more traditional forms of research, to be considered ethical, potential participants and communities should be involved early on in a ‘meaningful participatory process’ (9 Guideline 7). Citizen science provides a particularly striking opportunity to rethink questions of fairness in knowledge production. The stated aspirations and advantages of many citizen science initiatives are of ‘open,’ ‘accessible’ and ‘citizen-driven’ participation. Yet claims that citizen science will lead to ‘better’ outcomes compared with ‘traditional’ forms of scientific knowledge creation require empirical investigation.^32^ Participatory processes are fraught with power imbalances between researchers and participants,^33^ in particular when working across socioeconomic gradients to ensure benefit to participating communities^34^ or with marginalised groups.^35 36^ In particular, medical research projects that uncritically promote public or patient ‘engagement’ have failed to create reciprocal and mutually beneficial relationships.^37^ As was shown in community inclusion efforts in South Africa, for example, the mode of approach can be as important as the content of the intervention.^38^ Thus, in line with guidance on ethical research in potentially vulnerable communities, (9, Guideline 7) the specific meaning of terms like ‘community’ or ‘public engagement’ within the context of a given project should be made explicit in the stated goals and invitations to participate.

Further, knowledge production is always embedded in specific social, political and institutional contexts. But arguably, types of knowledge production that prominently draw on the work and ingenuity of non-professional experts represent a particular opportunity—and perhaps also responsibility—to foreground questions of equity and justice. As scholarship within feminist science studies has shown, questions of equity and justice are built into the design of things as fundamental as lab meetings, including how speaking time is allocated and how decisions are reached.^39^ Scholarship addressing inequalities in specific political contexts has developed recommendations for research with historically marginalised groups, including the imperative of studying *with* members of Indigenous groups as full research partners who develop questions, own data and get paid for labour.^40^ Such guidelines build on a growing body of work in indigenous research methodologies,^41^ and efforts to recognise concepts of reciprocity or place in empirical research.^42^ Drawing from this, critical reflection on participation begins with an analysis of power differentials: How does a project proactively address disparities in position, access, experience or resources? What explicit codes of conduct do participants agree on, and how are they determined? Attention needs to be paid to lay participant engagement, and reciprocally to the quality and depth of the involvement of researchers themselves—reflecting particular obligations raised for those designing, running and funding citizen science initiatives.^37^

Much of the expansion of citizen science has been facilitated by digital technologies, in particular smartphones, wearables and the internet. Yet, the accessibility of health data and digital technologies hinges closely on persistent socioeconomic inequalities.^43 44^ Access must be considered multidimensionally, in terms of geography, language, skills, time and tools: How do different groups engage with the technologies being used in the project? Are participants merely ‘consuming’ web content, or are they also able to alter or provide content? Do website descriptions include visual aids and multimedia pieces that explain in plain language the aims of the project, and how to participate? What forms of literacy (scientific, medical, digital, English language) are required? Iterative reflection on how these technologies and their modes of access (eg, websites, apps, smartphones, wearable devices) might exclude certain groups, and how this could affect the evidence that is produced, becomes critical to any project aiming to improve access to health resources, in particular for disadvantaged participants.

Monetary aspects deserve special consideration as a barrier to participation. Unlike in more traditional forms of research, where participants should be reasonably reimbursed for their costs and compensated for their inconvenience and time (9, Guideline 13) many citizen science initiatives rely on financial contributions from participants. Such contributions can be solicited either directly as a service or membership fee, or indirectly through the obligation to possess software or tools and could form obvious passage points that exclude certain groups. This practice shapes the resulting data set: only people who can afford to do so have their guts sampled and analysed, for example. Projects that use a consumer-as-participant model have been found to employ a more limited kind of participation,^45^ and financial conditions can compound other barriers such as computer access or time availability, raising the concern that citizen science might remain an upper class and largely white phenomenon.^46^ The questions of why fee-for-participation models are accepted within some projects, and when it would be considered both unusual and problematic in traditional research (9 Guideline 13) deserve more systematic probing and public debate.

The shift towards wider participation in science creates benefits, and it can affect the distribution of direct and indirect burdens and responsibilities borne by participants. In the area of medicine, public participation is changing what it means to be an informed and active patient. One of the most powerful cultural shifts that has accompanied participant-driven genetic research has been growing social expectations that individuals should monitor and manage their own health.^19^ Particularly where the prevention and management of disease is framed as primarily an individual responsibility and not a collective duty, the possibility exists that citizen science could become a smokescreen for the retreat of public service-provision. Likewise, the uncritical celebration of participation could lead to the stigmatisation of non-participation, disadvantaging those who do not participate in self-tracking, data sharing or community research. We share the worry of Juengst *et al* 47 that in times of increasing budgetary pressures, healthcare systems could set priorities in favour of the ‘empowered patient.’ It is thus imperative that participatory projects remain completely voluntary and not come to replace other, publicly funded means of accessing treatment and care.

## Public goods, benefits and distribution of results

Many citizen science initiatives appeal broadly to the advancement of science, medicine and the public good. Websites encourage individuals to contribute for the good of ‘the network’^26^ or to address global problems. Vague encouragements to ‘Explore your gut and help science’ (UBiome) or calls to ‘donate your data, for you, for others, for good’ (PatientsLikeMe), contemplate benefit in generic terms. What are the ethical implications of calling on prosocial motivations to entice participation without specifying the intended collective benefits^48^ and how will they be delivered? As a condition of research ethics review in more traditional forms of research, the scientific and social values of the intended research initiative must be specified; these values can never override the rights of research subjects (9 Guideline 1). Further, any research initiative must have an equitable distribution of benefits and burdens for participants (9 Guidelines 3 and 4, 10). If citizen science initiatives are to avoid exacerbating inequalities in biomedical research, the benefits and harms of such projects need to be specified in greater detail. Delimitation of specific benefit would include anticipating who could be empowered and disempowered in the process. As practitioners working in public health have shown, concrete steps such as community advisory groups can help hold researchers accountable.^33^

Many citizen science projects seek to develop a new tool, product or database for scientific use, which often includes the solicitation of contributions (time, biosamples, personal information or knowledge) from patients or other citizens. For project leaders and researchers seeking to promote equitable access of prosocial products, tools and information, the following questions will be relevant: How can we make our products of better service, in particular those who need it the most? Several initiatives in the vein of ‘DIY’ science make public dissemination their explicit goal. In this spirit, projects in medicine, genetics and public health could consider how their tools, databases, techniques and therapies can be made widely available.

Finally, advances in digital media, portable devices, open-source databases and social networking have broadened both the scope and speed by which publics can access and mobilise their own medical data and contribute to scientific knowledge production.^49^ A common assumption in the discourse on citizen science is that access to one’s own information leads to the democratisation of research and healthcare.^4^ Claims of democratisation generally envision a world in which patients have full access to their own data as well as medical technologies and are thus able to take on a far more proactive role in managing their health and well-being.^4 50^ While it is doubtlessly true that data may empower some, claims of democratisation merely via providing technical access to data have not been sufficiently demonstrated.^16^ Data access policies mirror socioeconomic inequalities within an assumed egalitarian landscape.^51^ Retaining a copy of individual-level data does not necessarily make the respective initiatives or society more democratic—although it can be an important step in this process. If, and how, access to uninterpreted data in citizen science can address matters of injustice and representation remains open for further consideration.^52^

## Representation and recognition

The ‘opening’ of the research process in citizen science raises new questions over representation, and has not been systematically addressed by established frameworks of research ethics within the context of health, biomedicine and participant-led practices. The selection of an appropriately representative research population has been a central, although significantly contested, research aim for scientific studies.^53^ Beyond scientific concerns, there are compelling ethical and political concerns surrounding the composition of a sample population. Non-representative study cohorts make it harder for some groups—such as women, people of colour, the elderly—to get good medical care.^54^ Particularly notorious in genomics,^55^ genome-wide association studies underrepresent African, Latin American, indigenous populations.^17^ This can result in personalised medicine being a tool of the few.^47^ At the same time, markers of racial difference often conflate socially constructed racial classifications with genetic diversity, thereby re-inscribing race in medicine and science in troubling ways.^56^ While the problem of representation is common to biomedical research, we focus on genetic and personalised medicine in order to address issues specific to recruitment and representation in citizen science.

Citizen science initiatives that are invested in the scientific search for diversity, such as in human gut flora or DNA, raise specific concerns.^57^ One strain of citizen science aims to build large, public data sets (American and British Gut Projects). Others, like the Genographic Project seek to catalogue the DNA of ‘unique’ populations, in order to ‘invite, encourage, and educate the public through participation in this real-time citizen-science project’ (National Geographic).^58^ The solicitation and use of indigenous genetic samples is inherently problematic given histories of colonialism, fetishisation of indigenous bloodlines and potential for profit.^59^ Challenging ‘collaborative’ projects like Genographic,  Tallbear argues that the interests served are not the ‘traditional’ groups they sample from. Efforts to be genetically inclusive need to attend to long histories of colonial violence that inform the mapping of indigenous genomes. One immediate result that flows from this is that citizen science projects of this nature need to be codirected by the groups being studied.^60^

Given that a swath of initiatives is dedicated to building public data sets, often including genetic analyses or advancing knowledge of rare diseases, the problem of representation is in need of explicit and continuous attention in order to ensure that assumptions about race are not re-inscribed in science, that the involvement of indigenous peoples in research is only as codirectors, and at the same time, that underserved groups are adequately considered in the search for medical interventions.^61^ Which groups are represented in a project is an important question that needs to be discussed by and with (potential) participants, remaining open to revision throughout the project.

It is important to recognise the strong historical roots of citizen science within community-based and participatory health research, often in response to persistent health disparities and legacies of medical racism. Long before such efforts were called ‘citizen science', marginalised groups took health research into their own hands, employing alternative modes of empirical inquiry, contesting authority and pursuing public collaborations.^62 63^  A branch of emancipatory citizen science initiatives follows in this vein (eg, Ciencia Forense Ciudadana). Yet, if citizen science of health is to strike a course divergent from traditional biomedical research, initiatives need to reckon with histories of racial and colonial violence that are entangled in the institutions of science, medicine and the state. It remains a pertinent question if, and how, contemporary citizen science can offer a means of addressing historical marginalisation.

## Trust, risk and global inequalities

Trust is an important factor in all forms of medical research (9 Guideline 7). However, it is arguably of even higher importance in contemporary citizen science; many people would not participate in a project or initiative without trust in the organisation or institution running it, as well as trust in fellow participants. Trust has been a perennial problem for biomedicine, as discussed, for example, in biobanking, organ donation, participation in clinical trials and elsewhere.^64 65^ By involving lay people as ‘collaborators,’ citizen science has been proposed as a tool to bolster waning enrolments in biomedical studies,^6^ to increase research population diversity and to galvanise lay participation.^66^ The involvement of members of marginalised groups in the development of research aims and recruitment strategies could open possibilities for addressing issues of exclusion in medicine. However, while there are compelling arguments to be made for the benefits of inclusive participation in research on the whole, and for promoting trust and transparency, discussion of different groups’ varying involvement in citizen science (whether distinguished by race class, gender, geographical location, education, etc) needs to be probed not just as an issue of differing degrees of ‘interest,’ but as situated within pertinent histories of marginalisation.^67^

Questions of trust are especially relevant in citizen science because, unlike conventional medical research, some citizen science initiatives—if they are led by patients and take place outside of established research institutions—do not undergo established risk-benefit analysis by internal review boards or research ethics committees.^30^ While this can enable fast, non-bureaucratic research on issues of social import, it also brings problems. The risks of citizen science initiatives are often uncertain because many projects chart new territory in real time reporting of disease, genomic research or experimental therapies. The range of ethical concerns might widen along with new collaborative opportunities, particularly across borders. We can imagine situations where the collection and analysis of samples, reporting of real time events or sharing of personal data could pose personal harms to individuals while at the same time offering important insights of public health benefit. One example would be a farmer who risks losing her flock when reporting signs of avian influenza.^68^

Potential harms that might be inherent to research are amplified along socioeconomic gradients and are especially relevant for marginalised, resource-poor populations. The inclusion of lay researcher populations from less developed regions make this an important concern for citizen science initiatives, particularly those not mandated to undergo ethics review. The development of strategies to avoid or mitigate harm with an eye on global inequalities, as well as wider access to research ethics consultations in participatory health practices, would be a strong step forward.^3^

## Conclusion

The incorporation of citizen science into new areas of health research potentially promises significant health and societal benefits.^18 69^ However, it also engenders concerns that have not been fully addressed by scholars, practitioners and funders working in the areas of health and medical research. We have argued that greater attention needs to be paid to the discursive and material exclusions that occur for underserved populations in participatory forms of health research. Given that many initiatives make an explicit claim to inclusion, it would be reasonable to expect meaningful participation in such initiatives, and that resulting benefits are equitable. This means that benefits need to be both accessible and actionable by participants—in particular those who are resource-poor, located outside of service networks, non-white/Western or have been historically marginalised by biomedicine.

This does not mean advocating a tokenistic approach to inclusion that essentializes notions of race or gender, and overlooks how data collection is entangled with histories of colonisation and exclusion. Instead, and in addition to existing guidance on ethical research practices in health and medicine,^9–11^ we argue for the creation of specific, proactive steps for the inclusion of various peoples and publics, which will vary with the goals, needs, settings and historical precedents of individual projects. As a first step, we have compiled questions that those designing, running, funding, participating in or providing ethical oversight for citizen science projects could consider in table 1. These recommendations are intended contextually; each project should engage with the concerns most relevant to collaborators.

Many citizen science projects have been made possible by advances in tools and strategies to collect, store and analyse digital data that are transforming global and public health. Hailed for their affordability, real time data and greater reach, such technologies also imply ethical obligations, especially when they involve the collection of samples and data from underserved populations. If citizen science initiatives are to fulfil their potential of serving the public good, they should address a diverse set of needs, and should do so in a way such that the collection and analysis of data are informed by the interests of those involved. We advocate that these concerns be built into the very design of citizen science and participatory health projects, rather than be treated as retrospective constraints. Given the attention participant-centric initiatives often command, there is great potential to engage in public reflection on questions of cultural relativity, standards of privacy, data ownership and accountability that are persistent concerns in medicine, bioethics and public health.
